# Femoral Pathological Fracture as the First Clinical Manifestation of Papillary Thyroid Carcinoma in a Primigravida

**DOI:** 10.1155/2013/397361

**Published:** 2013-04-04

**Authors:** Ahmed Abu-Zaid, Ayman Azzam, Hindi Al-Hindi, Tarek Amin

**Affiliations:** ^1^College of Medicine, Alfaisal University, P.O. Box 50927, Riyadh 11533, Saudi Arabia; ^2^Department of Surgical Oncology, King Faisal Specialist Hospital and Research Center (KFSH&RC), P.O. Box 3354, Riyadh 11211, Saudi Arabia; ^3^Department of Pathology and Laboratory Medicine, King Faisal Specialist Hospital and Research Center (KFSH&RC), P.O. Box 3354, Riyadh 11211, Saudi Arabia

## Abstract

Papillary thyroid carcinoma is the most common differentiated type of thyroid malignancy. It is largely a loco-regional disease with a high tendency to metastasize to regional cervical lymph nodes. Distant hematogenous metastases are very rare and primarily include lungs and bones. Distant bone metastases are present in approximately 1.7% of patients with differentiated thyroid malignancy. Sternum, ribs, and spine are the most frequent sites of osseous metastases. Up to our knowledge, we report the first occurrence of an extra nodal metastasis of papillary thyroid carcinoma to a femoral bone presenting as a pathological fracture in a 21-year-old 37-week primigravida. We report this case because of its unusual site of metastasis and atypical presentation during pregnancy. Moreover, we briefly elaborate on the management of such uncommon cases.

## 1. Introduction

Papillary thyroid carcinoma (PTC) is the most frequent differentiated histological form of thyroid malignancy accounting for approximately 80% of all primary thyroid cancers [1]. PTC is largely a localized disease confined to neck area with a high propensity to metastasize to regional cervical lymph nodes [2]. Hematogenous distant metastases from PTC are extremely rare [3]. At the time of diagnosis, bone metastases are present in less than 2% of all patients with differentiated thyroid malignancy [4]. Sternum, ribs, and spine are the most common locations of bone metastases [5]. Herein, we report an extra nodal metastasis of PTC to a femoral bone presenting as an incidental pathological fracture in a 21-year-old 37-week primigravida.

## 2. Case Report

A 21-year-old 37-week primigravida was referred to our hospital with a femoral pathological fracture following a minor fall event at home. The patient was known to have goiter for three years. The goiter was predominately left-sided and stable with neither significant increase in size nor obstructive symptoms. Exposure to ionizing radiation and family history for thyroid malignancy were negative. Systemic review was remarkable for a one-week history of on/off back pain. The course of pregnancy was uneventful.

Upon admission, laboratory work-up was significant for serum thyroglobulin (TG) of 1,368.0 *μ*g/L (normal range: 1.4–78.0 *μ*g/L). Neck ultrasound (US) was done and showed a unifocal 11.7 × 7.1 × 8.2 cm left-sided heteroechoic lesion involving the left thyroid lobe with moderately increased vascularity, foci of calcifications, and areas of necrosis. The neck US was highly suggestive of primary thyroid malignancy with no suspicious lymph nodes. Fine needle aspiration of the suspicious neck lesion confirmed the diagnosis of PTC. The patient was admitted for pregnancy delivery and further tumor work-up after delivery.

Day-7 after admission, the patient delivered her baby at 38 weeks of gestation by an uneventful lower segment cesarean section. The baby was otherwise healthy and placenta was not examined histologically for search of metastasis. After delivery, the patient was worked up for her femoral pathological fracture and query of metastatic PTC. Computed tomography (CT) scan showed no evidence of lung metastasis or lymphadenopathy. Magnetic resonance imaging (MRI) scan showed destructive expansile osteolytic lesions involving the left proximal femur, the vertebral body of L2, and the right transverse process of T10. A multidisciplinary team was involved in the management of the patient.

**Figure 1:**
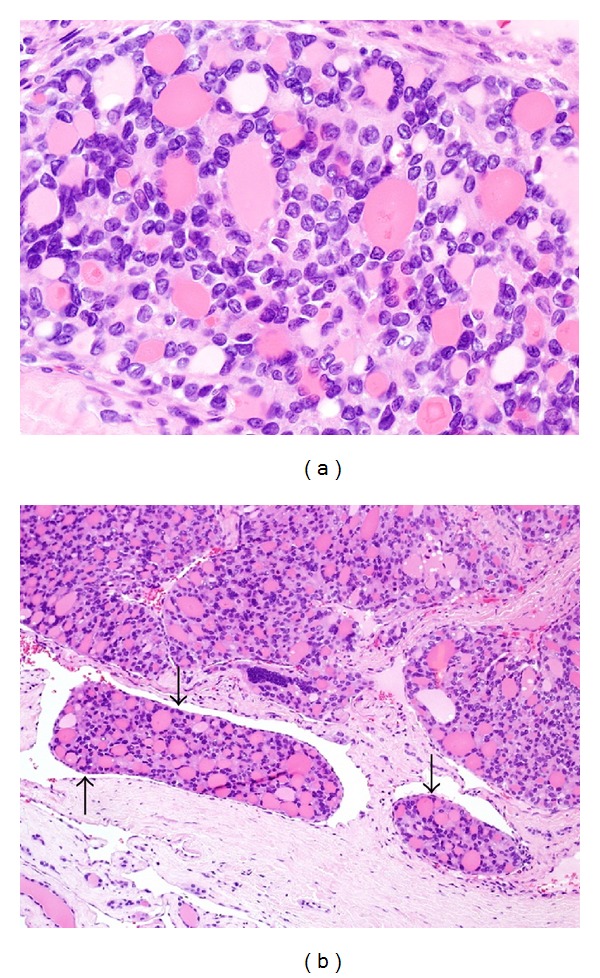
Histopathological examination of the left thyroid lobe showing papillary thyroid carcinoma (H&E stain). (a) Tumor cells form follicles lined by cells with typical nuclear features of papillary thyroid carcinoma of crowded enlarged nuclei with prominent nuclear grooves. (b) There are several foci of vascular invasion. Note the endothelial lining of tumor emboli (arrows).

**Figure 2:**
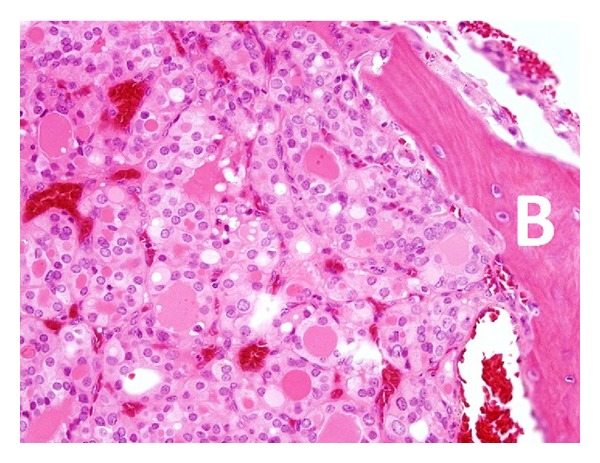
Histopathological examination of the resected left femur showing a follicular pattern of papillary thyroid carcinoma (H&E stain). A bone spicule (b) is noted on the right side.

Patient underwent total thyroidectomy with left and central neck dissection. Grossly, the tumor was well circumscribed measuring 9.5 cm in the maximum diameter showing vascular invasion and without signs of invading surrounding tissues. Histopathological examination of the left thyroid lobe was consistent with primary PTC (Figure 1). All the twenty-nine reactive lymph nodes identified in the neck dissection specimen were negative for metastasis. Moreover, during the same surgery, the patient underwent left proximal femur resection with endoprosthesis. Histopathological examination of the resected proximal femur was consistent with metastatic PTC (Figure 2). Three months after left femur implantation, patient developed severe chronic prosthetic infection for 4 months which was managed aggressively with antibiotics. Two weeks after surgery, the patient underwent L2 vertebral body resection with posterior decompression (foraminotomy), interbody fusion, stabilization, and instrumentation. Histopathological examination of the resected L2 vertebral body was consistent with metastatic PTC. The surgical resection of the T10 mass was not a favorable option for three reasons: first, positive history of chronic left femur prosthetic infection; second, increased risk of metal chronic infection; third, the bloody nature of T10 tumor with possible life-threatening operative complications. Instead, the plan was to consider the patient for ten in-patient spine external beam radiation therapy (EBRT) sessions spread over two weeks without any further surgical intervention.

Postoperatively, patient had a single session of radioactive iodine (I-131) therapy (dose: 190 mCi). A follow-up US of neck soft tissue showed a clear right thyroid bed and a hypoechoic nodule in the left thyroid bed measuring 0.4 cm in the maximum diameter with no vascularity and no suspicious lymph nodes. Postoperative thyroid function tests (TFTs) showed normal values and TG dropped to 51.6 *μ*g/L (normal range: 1.4–78.0 *μ*g/L). Whole body positron emission tomography/computed tomography (PET/CT) scan showed interval resolution of neck findings with postoperative changes and multiple metastatic minimal fluorodeoxyglucose (FDG) avid bony lesions around the left total hip replacement prosthesis, L2 and T10. The patient was scheduled to be seen after 3 months.

## 3. Discussion

PTC is the most common type of thyroid malignancy encountered in endocrinology clinics [1]. PTC can occur as a part of a familial syndrome (3%) or sporadically (97%) [6]. Exposure to ionizing radiation significantly increases the risk of developing PTC [7]. Although multifocality [8], bilaterality [9], and local lymphatic metastases [2] are very common features in PTC, interestingly, our patient had an unusual presentation of a unifocal and unilateral malignant lesion with no regional lymph node metastases at the time of diagnosis.

PTC, as a largely localized slowly growing disease with an indolent clinical course, has a favorable prognosis and much lower cancer-related mortality rate than other malignancies [10]. Young age (less than 45 years) greatly influences staging and is a very significant positive prognostic factor in thyroid malignancy [3]. The presence of distant PTC angioinvasive metastases at the time of diagnosis is exceptionally rare and adversely affects prognosis and survival rate [11]. Distant bone metastases are present in approximately 1.7% of patients with differentiated thyroid malignancy [4] with the sternum, ribs, and spine being the most frequent sites of osseous metastases [5]. Distant metastases from primary PTC to femoral bones are broadly uncommon. Up to our knowledge, this is the first reported occurrence of an incidental femoral pathological fracture exhibiting metastatic PTC in a pregnancy setting. This suggests that PTC metastasis to an exceedingly uncommon site, such as a femoral bone with a resultant pathological fracture, may be the first manifestation of an incidental primary PTC presenting at the time of diagnosis.

The presence of multiple extra nodal metastases of an advanced primary thyroid malignancy in the setting of a possibly tumor-aggravating factor such as pregnancy [12] presents a complicated management decision. Pregnancy may act as an aggravating factor in primary thyroid carcinoma. This is probably presumed via the role of human chorionic gonadotropin (*β*-HCG) hormone which may bind to the TSH receptor and stimulate further tumor proliferation [12]. Only less than 3% of pregnant women with PTC present with distant metastases at the time of diagnosis [13]. Up to our knowledge, the presented case report discloses the first occurrence of a femoral pathological fracture presenting in a pregnant woman with PTC.

A well-judged management of such cases requires consideration of three issues. First, postponement of the surgical intervention of PTC diagnosed late in an uneventful pregnancy: the prognosis of primary or metastatic thyroid malignancy in pregnant and nonpregnant women does not seem to be dramatically different, and the surgical intervention of primary or metastatic thyroid carcinoma diagnosed late in the course of pregnancy can be postponed safely until after labor [13]. Second, consideration of extensive thyroid surgical clearance (total thyroidectomy and radical neck dissection) followed by ablative radioactive iodine (I-131) treatment: the mainstay treatment of differentiated thyroid malignancy remains surgical resection followed by ablative I-131 and postoperative thyroid-stimulating hormone (TSH) suppression. Third, consideration of instantaneous resection of operable solitary bone metastases with or without subsequent external beam radiation therapy (EBRT): the use of I-131 in the treatment of metastatic PTC bone lesion is not effective (suboptimal) and does not yield complete response [14]. Conversely, the use of EBRT has been strongly suggested in the management of loco-regional gross/microscopic residual disease after surgical resection [15] as well as in the management of inoperable bone metastases in patients with PTC [16].

Successful management of such complex clinical cases requires involvement of a multidisciplinary team and such patients should be thoroughly investigated and followed up for cancer recurrence.

## 4. Conclusion

To the best of our knowledge, we report the first occurrence of an extra nodal metastasis of PTC to a femoral bone presenting as a pathological fracture in a pregnant woman. Metastasis of thyroid carcinoma should always be considered in the differential diagnosis of pathological fracture in any patient presenting with a history of thyroid malignancy, and histopathological examination of the bone specimen is essential for confirming the diagnosis. Successful management of such complex clinical cases requires involvement of a multidisciplinary team.
